# Pregnancy in Patients with Inflammatory Bowel Diseases—A Literature Review

**DOI:** 10.3390/life13020475

**Published:** 2023-02-09

**Authors:** Raluca Roxana Grigorescu, Ioana Alexandra Husar-Sburlan, Georgiana Rosulescu, Anca Bobirca, Razvan Cerban, Florin Bobirca, Madalina Marieta Florescu

**Affiliations:** 1 Gastroenterology Department, “Sfanta Maria” Hospital, 011172 Bucharest, Romania; 2 Department of Internal Medicine and Rheumatology, Dr. I. Cantacuzino Clinical Hospital, “Carol Davila” University of Medicine and Pharmacy, 020021 Bucharest, Romania; 3 Center for Digestive Disease and Liver Transplantation, Fundeni Clinical Institute, Carol Davila University of Medicine and Pharmacy, 020021 Bucharest, Romania; 4 Surgery Department, Dr. Ion Cantacuzino Clinical Hospital, 011437 Bucharest, Romania

**Keywords:** inflammatory bowel disease, pregnancy, breastfeeding

## Abstract

In recent years, we have faced an increasing incidence of inflammatory bowel disease (IBD), especially among young people, affecting them during their reproductive years. The paucity of data and reduced knowledge regarding the evolution of the disease during pregnancy and the adverse effects of the therapy on the mother and infant increase voluntary childlessness in this group of patients. Depending on the type of IBD, severity and surgical or medical management, this can negatively affect the pregnancy. C-sections and the risk of low-birth-weight babies are higher in women with IBD, independent of active/inactive disease, while preterm birth, stillbirth and miscarriage are associated with disease activity. In the last period, medicinal therapy has evolved, and new molecules have been developed for better control of the lesions, but the effect on pregnancy and breastfeeding is still controversial. We conducted this review by studying the literature and recent research in order to have a better image of the practical management of IBD during pregnancy.

## 1. Introduction

Inflammatory bowel diseases represent a group of chronic inflammatory conditions that predominantly affect the digestive tract. They are composed mainly of ulcerative colitis (UC) and Crohn’s disease (CD) [1,2]. While CD can affect any segment of the digestive tract in a segmental, asymmetric and transmural manner, potentially causing the appearance of complications such as strictures, fistulas or abscesses, UC is typically characterized by mucosal inflammation that begins in the rectum and can extend continuously to the proximal colonic segments. At present, there is no curative treatment for these diseases, both of which require long-term treatment to control symptoms and reduce the risk of complications.

The incidence of IBD has increased in the last few years [3]. For women, the mean age at diagnosis for CD and UC is 26 years and 32 years old, respectively [4]. This coincides with a large proportion of their reproductive period, with 25% of female patients having their first pregnancy after diagnosis [5].

Compared with the general population, the fertility rate in women with IBD is slightly lower, which was estimated in a large cohort study to be 46.2 live births per 1000 person-years [5].

Patients generally have poor knowledge about pregnancy-related topics, which can lead to multiple concerns about their fertility and the impact of pregnancy on the disease itself and on the offspring [6,7], and higher voluntary childlessness correlated with lower education levels [8].

On the other hand, obstetricians/gynecologists expressed discomfort with the initiation of IBD medications around conception and pregnancy period, which might result in under-treatment of IBD flares and, subsequently, poor pregnancy outcomes [9]. 

The scarce data available about pregnancy-related problems in IBD patients led us to research this topic in order to systematize the information available until now on the prenatal, pregnancy and breast-feeding period for a better understanding and optimal guidance for both patients and caregivers on the management of the disease during this special period. 

In some countries, there are dedicated IBD clinics that have IBD gastroenterologists, obstetricians experienced in IBD management, dietitians, psychologist and colorectal surgeons that can offer education about risk reduction, fertility, medication management and delivery options, [10] leading to a higher pregnancy rate and less voluntary childlessness [8].

## 2. The Influence and Involvement of IBD in Reproduction

Infertility is the inability to conceive within 1 year of unprotected sexual activity, according to the World Health Organization [11]. IBD patients are frequently under 35 years at diagnosis [12]; therefore, this disease and treatment may have a great impact on reproduction, fertility and sexual health. In one study, almost a quarter of patients with IBD (26.7% with CD and 23.3% with UC) report sexuality impairment [13]; this problem is often not addressed by medical personnel [14], although over half of the patients expect to discuss this topic with a physician [15]. 

Fertility in IBD may be decreased by many factors, such as flares of disease, psychological factors, malnutrition, or medical and surgical treatment. It was reported that patients during remission have the same prevalence of sexual dysfunction as controls. However, during a flare, patients tend to have severely impaired sexual function due to depression [16,17] that may interfere with testosterone secretion causing impairment of spermatogenesis [18,19], erectile dysfunction [20] and lower libido [21,22]. 

The rate of voluntary childlessness (VC) is increased among patients with IBD, about 18% in CD, and 14% in UC vs. 6.2% in the general population [23], mainly due to misperceptions about pregnancy and their disease. The desire to have children is affected by fear of relapse or complications that may interfere with the ability to care for a child, congenital abnormalities, transmitting IBD and teratogenicity for both women and men [24,25,26]. A survey that included 1.324 women with IBD reported poor disease-specific pregnancy knowledge as the leading cause of VC. Thus, it is of great importance to counsel these patients through a family planning program and provide correct information regarding the risks associated with pregnancy. In fact, it was noticed that female patients that have received specialized preconception, intrapartum and postpartum counseling had improved maternal and fetal outcomes [27,28], but few patients have access to specialized clinics. Therefore, in most cases, websites and information sheets such as https://ibdpregnancyaid.com/ (accessed on 31 January 2023) can help patients to make a correct decision together with their healthcare providers [29,30,31]. 

Regarding genetic transmission, an increased risk was observed in CD compared to UC (2.7% vs. 1.6%) and can exceed 30% if both parents and other family members have IBD [32]. If only one parent is affected by CD or UC, the relative risk of developing IBD is 6–7.5%, respectively, four-fold higher than for a child whose parents do not have IBD [33]. The risk is even greater if the mother is affected by CD and if the offspring is female [34,35]. If a child has more than two affected first-degree relatives, the risk is increased to 9.77% for CD and 6.63% for UC [33]. 

The IBD Parenthood Project Working Group launched by the American Gastroenterological Association (AGA) recommends 3–6 months of clinical remission before conceiving, 6 months after withdrawal of a teratogenic drug and 6 months after withdrawal of any experimental drug. Thus, in times of flares or severe activity disease, contraception is an important part of family planning. Intrauterine devices and implants are the first-line recommendations, although all the other forms of contraceptives are acceptable [24].

### 2.1. The Influence of IBD on Reproduction in Women

In two population-based studies, the fertility rate was slightly lower in women with IBD (46.2 live births per 1000 person-years) compared to the general population (49.3 live births per 1000 person-years). Depending on the type of IBD, it was observed that patients with CD and those with IBD that required bowel resection had a lower fertility rate, while those with UC who did not require surgical intervention had a fertility rate similar to the general population [5,36]. The fertility during flares of disease is decreased to 35.6 live births per 1000 person-years regardless of the IBD type [5]. The active disease could impair fertility through local inflammation involving reproductive organs, depression, malnutrition and anemia [24,37].

Sexual dysfunction can lead to lower fecundity rates. IBD female patients report a lower sexual quality of life [38]. In an Australian survey, it was found that over three-quarters of female patients with IBD, especially those who have undergone surgery, have an impaired body image, and over half have a decrease in libido and the frequency of sexual activity [22]. Women with CD have more difficulty achieving orgasm, increased dyspareunia and deep dyspareunia compared to controls [39].

Oral contraceptives have been reported to be a risk factor for developing IBD [24]. Despite the factors that decrease fertility, female patients had an increased use of contraceptive methods suggesting a higher rate of voluntary childlessness (VC) [5,23,40]. Studies have found that 19–37% of IBD women are voluntarily childless [38,41].

Some studies reported that females with CD have a decreased ovarian reserve as measured by serum anti-Müllerian hormone and an accelerated risk of losing fertility with age, especially beyond the age of 30 [40,42,43,44]. The mechanisms may be both direct by inducing inflammation of the fallopian tube or indirect due to tubal adhesions after surgical interventions [43,44].

Assisted reproductive technology (ART) may be an option for women with infertility and IBD. A recent meta-analysis by Laube et al. concluded that ART is safe and effective for patients with UC and CD medically managed, with results similar to the general population, but with reduced efficacy for women with CD-related surgery and IPAA (ileal pouch anal-anastomosis) failure [45].

Regarding treatment, mesalazine, corticosteroids, thiopurines and anti-TNF have not been shown to impact fertility in women [46], while methotrexate and tofacitinib, although they may not impact fertility in women, are contraindicated during the preconception period because of their teratogenic effects [47,48]. Ustekinumab and vedolizumab data are lacking.

When speaking about IBD, we have to mention the high rate of surgical interventions. Statistics show that in 10 years, 50% of CD and 15% of UC patients require surgery. The results concerning the influence of surgery on fertility are weak and of low evidence [49]. A systematic review noted that infertility, stillbirth and preterm birth are not associated with a history of abdominal surgery, but there is a slight association with miscarriage, low birth weight and cesarian section [50]. A recent systematic review and meta-analysis that compared fertility in women pre and post-ileal pouch anal-anastomosis (IPAA) showed a relative risk of infertility of 4.17%. This is as high as the one noted decades before, despite the new surgical techniques [51]. No statistical difference was observed in the infertility rates for women who have open or laparoscopic IPAA [37]; however, ECCO recommends the laparoscopic approach [36]:

- It has been shown that in CD, depending on disease activity, duration and extent of the disease, malnutrition can affect up to 85% of patients [52,53]. Micronutrients deficiencies may affect fertility through different mechanisms;
- Folate deficiency may impair oocyte quality, maturation, fertilization and implantation;
- Zinc may alter the menstrual cycle by affecting ovulation, fertilization, normal pregnancy, fetal development and parturition [54];
- Vitamin A may impair oocyte quality and blastogenesis [54];
- Vitamin D is involved in estradiol and progesterone production [55]. A sufficient preconception level of vitamin D is associated with an increased likelihood of pregnancy and live birth [56] and a higher rate of ART success [57];
- Vitamin B and iron are involved in embryogenesis and homocysteine metabolism [58,59]. Normal levels of iron are associated with a lower risk of ovulatory infertility and a decreased risk of adverse birth outcomes [60].

ESPEN guidelines recommend that patients with IBD should be checked regularly for micronutrient deficiency [61], and increased attention should be taken into account when the patient wants to conceive.

### 2.2. The Influence of IBD on Reproduction in Men

Infertility in men with IBD is not well studied, although it is believed to be higher in this group [62]. As in the case of women with IBD, it was observed that men with CD have a lower number of children compared to patients with UC or the general population [63,64], even though during remission, the frequency of sexual intercourse was not significantly different between the groups [65]. In men, disease activity and depression can have a detrimental effect on sexual function [66]. In some studies, erectile dysfunction was reported in 94% of men early in the course of the disease [67], but with the passage of time since the diagnosis, probably through the development of some coping mechanisms, sexual function seems to improve [66]. During active disease, almost half of the men feel sexually compromised [66], while clinical remission frequently is not associated with sexual male dysfunction [68]. 

Regarding the effect of medication on male fertility, it is known that 5-ASA, especially the sulfapyridine component of sulfasalazine, can produce reversible oligozoospermia [69,70,71,72]. In rare cases, mesalazine can have the same effect, although with an unknown mechanism [73,74]. An observational crossover study concluded that exposure to dibutyl-phthalate contained in some mesalazine formulations, such as Asacol, impaired sperm motility that lasted for longer than 4 months [75]. If a male patient wants to conceive is better to change sulfasalazine at least 3–4 months prior [76] with another 5-ASA compound. If he is still unable to conceive and maintains disease remission, he could also stop mesalazine.

The impact of corticosteroids and azathioprine (AZA) on male fertility is still debated. In studies on rats, a reduction in fertility was observed in both. In the case of corticosteroids, the reduction was observed without a change in the number or motility of spermatozoa [77], while in the case of AZA, the quantity and quality of the sperm were reduced. Studies on men concluded that they either have no impact [78] or may have a decrease in sperm concentration [64]. 

Methotrexate (MTX) can induce reversible oligozoospermia, altered sperm integrity [79,80,81] and erectile dysfunction [82,83,84], although some studies did not find any adverse effects on fertility [21,85].

Anti-TNF seems to have no impact on fertility, pregnancy or child outcome when used by males [86,87,88,89,90,91]. Newer biologics have scarce data regarding the impact on fertility, pregnancy and fetal outcomes. Vedolizumab does not impact sperm quality and DNA integrity, and the seminal plasma levels were 1% of the serum levels; thus, female exposure after vaginal absorption is thought to be negligible [92]. 

Nutrition

Malnutrition is more prevalent in patients with IBD [61] and can lead to zinc deficiency. This mineral has a role in spermatogenesis, and in maintaining optimal serum androgen levels, lower levels have been associated in multiple studies with infertility and oligozoospermia [93,94]. 

Surgery 

Some studies report an improvement in sexual function and quality of life after surgery [95,96]. In patients with UC that had rectal exclusion or IPAA, sexual dysfunction was seen at rates ranging from 3 to 25.7% [97,98,99,100], but in 79% of patients, this was restored using sildenafil [101]. 

In some cases, patients with IBD require a stoma. This can induce erectile dysfunction and may also have a psychological impact, such as a decrease in self-esteem and the fear of involuntary eliminating odors or intestinal content, which lowers their desire for sexual intercourse [66,102,103]. 

## 3. Impact of IBD on Pregnancy

It has been proven that IBD can have a negative impact on pregnancy, depending on the type of IBD, severity, the extension of the disease and the treatment received [32]. Frequently reported complications to include premature birth, small for gestational age (SGA), spontaneous abortion, necessary C-section delivery, low APGAR scores or ICU admission [104]. The risk for these complications is increased by disease activity during pregnancy [10,105]. 

### 3.1. Small for Gestational Age (SGA)

Women with IBD have a higher risk of giving birth to low birth weight or SGA babies [32]. Cornish et al. concluded in a meta-analysis that the risk is threefold increased for CD [106]. For UC patients, the studies are still inconsistent, the results varying between 0 and 19.5% risk of giving birth to an SGA child. 

### 3.2. Preterm Birth

A percentage of 9–18% of women with IBD are at risk of preterm birth, compared to the general population (5–9% risk of premature birth). The risk is elevated in both UC and CD patients and especially high in patients with active disease [107,108]. Cornish et al. showed that the risk is twice as high in mothers with CD compared to the control group [106]. In the case of patients treated with biological treatment, premature birth was found in 9% of the cases [109].

### 3.3. Low APGAR Score

The risk of a low APGAR score at birth is 1.5 times higher for women with IBD using corticosteroids for active disease compared to the general population. Moreover, it was observed that the risk of a low score is three times higher for those with CD and less significant for those with UC [110].

### 3.4. Cesarean Section and Impact of Delivery Mode on IBD Outcome

Many studies have shown that the incidence of C-sections is increased in IBD patients compared with the general population [111,112]. C-sections are performed slightly more often in CD than in UC (52% vs. 48%), with a history of perianal disease for CD and previous colectomy for UC being predisposing factors [113]. No difference was observed regarding the natural evolution of IBD according to the type of delivery, cesarean or vaginal [114,115]. In some studies, two-thirds of CD patients with active perianal disease presented an aggravation of symptoms after vaginal delivery, and in the case of those with IPAA, complicated vaginal delivery was associated with impaired pouch function [116]. ECCO states that a C-section is indicated in active perineal disease or active rectal involvement [36]. In the absence of IPAA or perianal disease, the decision between vaginal and cesarian delivery should be based on obstetric indication and patient preference. 

### 3.5. Pregnancy Effects on IBD Activity

Regarding the influence of pregnancy on the course of IBD, a 30% risk of disease reactivation during pregnancy was observed, similar to non-pregnant women [117]. Women who become pregnant during active IBD or in the preconception period are more predisposed to have a flare during pregnancy and in the postpartum period compared to those who become pregnant during remission [28,111,118]. For this, European Crohn’s and Colitis Organisation (ECCO) recommends that women be in remission for at least 6 months before trying to conceive [36]. The type of IBD plays a role during pregnancies, and some studies conclude that patients with UC have a higher risk of relapse during pregnancy and in the postpartum period than patients with CD [118,119], but others have found that females with the penetrating or structuring form of CD also have an increased risk of postpartum flare [120]. A history of a disease flare during a previous gestation may be a risk factor for disease activity in further pregnancies [118,121]. 

Up to a third of IBD patients present the onset or a flare of IBD activity in the immediate postpartum period, especially those who have active disease in the last trimester of pregnancy, stop biological therapy in the third trimester or de-escalate therapy after delivery [120,122]. There could be other factors that could contribute, such as the hormonal changes that occur during this period as well as changes in the circadian rhythm according to the requirements of the newborn, disturbing the secretion of melatonin, which has an anti-inflammatory, anti-oxidant role and improves the intestinal microbiome [123]. However, pregnancy, in general, has a positive effect on IBD, decreasing the risk of relapse compared to the years before pregnancy [124].

### 3.6. Disease Activity

Patients with active UC and CD had an increased risk of preterm birth, spontaneous abortion, LBW and SGA [110]. These adverse pregnancy outcomes may be due to nutrition and inflammation. During an active flare of the disease, nutrition and absorption can be severely impaired, and various studies highlighted the transgenerational impact of the maternal gastrointestinal system and dietary habits on pregnancy [125]. Active IBD during the first trimester is associated with decreased infant weight and height up to 6 months of age [126]. Inadequate gestational weight gain during pregnancy was found to be a strong independent predictor for adverse pregnancy outcomes such as preterm (2.5-fold increased risk) and SGA, independent of disease activity [127].

### 3.7. The Impact of Treatment of IBD in Men on Pregnancy Outcomes

Children born to fathers who are on 5-ASA treatment, including compounds containing sulfapyridine, do not seem to have an increased risk of malformations or adverse pregnancy outcomes [128,129], although some studies with small sample sizes found an increased risk of congenital malformation [63], spontaneous abortion [78] or stillbirth [130].

Regarding the use of AZA by men at the time of conception, a meta-analysis found no increased risk of malformation and adverse pregnancy outcomes [131], but older studies found an increased risk of pregnancy-related complications [132].

MTX is known for the teratogenic effect in women, but when used by men is not associated with a higher rate of malformation or spontaneous abortion [80,129,133,134], but some active metabolites can remain in cells after discontinuation, and most reviews recommend interruption for at least 3-4 months before conception [37,135]. While anti-TNF use is not associated with adverse pregnancy outcomes or congenital malformation, vedolizumab and ustekinumab seem to be safe, but the data are scarce [129].

## 4. Management of IBD during Pregnancy

### 4.1. Follow-Up during Pregnancy

Assessment of disease activity in IBD is performed by clinical scores, biochemical parameters, endoscopy with biopsies and radiologic studies. Clinical scores during pregnancy may be less reliable because some of the symptoms might also be pregnancy related [136].

Endoscopy in pregnant women is associated with an increased risk of preterm birth and small for gestational age [137]. Multiple studies concluded that, whenever possible, endoscopy should be performed in the second trimester of pregnancy, with the patient placed in a left lateral position to avoid vena cava compression; flexible sigmoidoscopy should be performed without sedation or bowel preparation [10,27,36].

Magnetic resonance imaging may determine fetal stress by tissue heating effects and high acoustic noise levels [138,139]. While the possible teratogenic effect of gadolinium contrast agent is unknown and should be avoided in the first trimester [27], iodine-based contrast agents used for computer tomography may affect the thyroid function or the skeletal development of the fetus [140].

Serum biomarkers such as hemoglobin, C-reactive protein (CRP) and albumin may variate during pregnancy and may not correlate with disease activity [141]:

- C-reactive protein levels: Julsgaard et al. reported a correlation between disease activity and increased CRP levels only during the second trimester [142], whereas two other studies demonstrated the correlation in all trimesters [143,144].
- Fecal calprotectin (FCP) appears to have higher levels in pregnant women with IBD, but the values are even higher in those with active disease. Three studies reported the overall FCP levels in the presence of disease activity during the gestational period. Julsgaard and al. showed that the median FCP was higher for those with clinically active disease than controls in the pre-conception period (765 µg/g vs. 0 µg/g), in the first, second and third trimesters (783 µg/g vs. 0 µg/g, 983 µg/g vs. 0 µg/g, respectively, 438 µg/g vs. 0 µg/g) and in the postpartum period (548 µg/g vs. 0 µg/g). Similar reports were also made by Huang et al. [145] and Kammerlander et al. [143].
- Intestinal ultrasound is a non-invasive, accurate investigation to determine disease activity, extent and complications of IBD that can be performed without any prior bowel preparation. The feasibility and accuracy rate in pregnant women with IBD was evaluated in multiple studies that included 148 pregnancies and concluded that it is an adequate tool that offers a non-invasive strategy to closely monitor patients [146,147,148].

### 4.2. Treatment

#### 4.2.1. 5-Aminosalicylic Acid (5-ASA)

A meta-analysis demonstrated that using 5-ASA compounds during pregnancy is not associated with an increased risk of spontaneous abortion (OR 1.14, 95%Cl: 0.66–2.01), preterm delivery (OR 1.35, 95%Cl: 0.85–2.13), stillbirth (OR 2.38, 95%Cl: 0.65–8.72) or congenital abnormalities (OR 1.16, 95%Cl: 0.76–1.77) [128].

Mesalazine has poor transplacental transfer and reaches low levels in the fetal circulation, opposite to sulfasalazine and sulfapyridine that traverse the placenta, and cord blood levels are the same as maternal serum levels [149]. 5-ASA is considered safe in pregnancy, except for the formulations that are coated with dibutyl phthalate (DBP), which can cause male urogenital and skeletal abnormalities in animals and dysregulation of thyroid and reproductive hormones in humans [150,151].

Sulfasalazine may impair folic acid absorption, so in pregnancy, it has to be associated with folic acid supplementation to be safe (>2 mg/day) [36,152]. In men, the sulfapyridine moiety of sulfasalazine can reduce sperm motility and count and may increase abnormal sperm forms [153], so males should be advised to cease sulfasalazine three months pre-conception [69].

#### 4.2.2. Corticosteroids 

Corticoids can traverse the placenta, where they undergo rapid metabolism into less active metabolites, reducing fetal exposure. Shorter-acting formulations, such as prednisolone and methylprednisolone, are more rapidly metabolized than dexamethasone [154]. The PIANO registry (pregnancy in IBD neonatal outcomes) found an increased risk of gestational diabetes (OR 2.8, 95%Cl: 1.3–1.6), low birth weight (OR 2.8, 95%Cl: 1.3–6.1), preterm birth (OR 1.8, 95%Cl: 1.0–3.1) and infant infection within 4 months after delivery (OR 1.5, 95%Cl: 0.9–2.7), but no increased risk of congenital abnormalities [155]. Corticoids in late pregnancy can determine, in rare cases, neonatal adrenal suppression, which requires prompt treatment, ideally in a neonatal intensive care unit [156]. Some studies have identified an increased risk of cleft lip and palate secondary to corticoid administration during pregnancy [157,158], but a larger study that included 2372 cleft cases found no association between maternal corticoid use and cleft lip and palate in offspring [159]. Budesonide, a new corticoid compound with higher first-pass metabolism and theoretically less fetal exposure [160], has a paucity of studies regarding safety in pregnancy, but it seems that it is safe to use during pregnancy [161]. 

#### 4.2.3. Thiopurines

Azathioprine and mercaptopurine traverse the placenta and may rich up to 5% of maternal levels in the fetal blood samples [162]. Multiple studies are considering this drug safe during pregnancy [131,163,164,165,166,167,168,169], although some small studies found an increased risk of preterm delivery but did not adjust the data to the disease activity [170] or neonatal anemia [171]. 

#### 4.2.4. Methotrexate

Methotrexate has teratogenic and embryogenic effects. It can cause neural tube defects, development delay, ileal perforation, abnormal facial features and skeletal deformation, spontaneous abortion and miscarriages [47,172,173]. Women taking methotrexate that desire to conceive should cease the medication and take a high dose of folic acid for a minimum of 3 months prior [174]. 

#### 4.2.5. Cyclosporine

Cyclosporine may be effective for avoiding colectomy during severe flares of UC during pregnancy [175,176], but exposure during pregnancy is associated with maternal hypertension, pre-eclampsia, spontaneous abortion, preterm birth and low gestational weight [177,178,179,180].

#### 4.2.6. Anti-TNF 

Infliximab (IFX) and adalimumab (ADA) are IgG1 anti-tumor necrosis factor (TNF) monoclonal antibodies, which are transferred across the placenta in the second and third trimesters of pregnancy. Some studies observed that maternal IFX levels increased during pregnancy while ADA levels remained stable [181]. Moreover, the levels of infliximab and adalimumab in the median cord blood, at term, exceed the maternal levels at a maximum of 197% and 153%, respectively [182,183], and are higher for infliximab even though the administration of the drug was ceased at the same gestation week [184]. This was also shown in a linear regression model that found that ceasing infliximab at week 24.6 and adalimumab at week 36.8 led to a cord blood level of 3 µg/mL [185]. 

Regarding the safety of anti-TNF use during pregnancy, there are some controversies, but multiple studies, including a recent meta-analysis [109,186], found that using anti-TNF treatment throughout all trimesters of pregnancy is not associated with an increased risk of adverse outcomes or congenital anomalies (Table 1). ECCO guidelines suggest stopping anti-TNF around gestational weeks 24–26 to minimize the transplacental transfer [36]. Toronto consensus and AGA inflammatory bowel disease parenting working group recommend continuation of anti-TNF treatment throughout pregnancy [10,27] in order to avoid a disease flare, only adjusting last dose timings with final infliximab infusion 6–10 weeks before the estimated date of delivery, and final adalimumab injection 2–3 weeks before delivery [10]. Regarding child immunodeficiencies, Guiddir et al. signaled that four children exposed to IFX during pregnancy developed transient neutropenia [187].

#### 4.2.7. Anti-Integrin

Vedolizumab is a humanized IgG monoclonal antibody that inhibits the binding of α4β7 integrin to mucosal vascular addressin cell adhesion molecule-1. During pregnancy, it was shown that maternal vedolizumab clearance is increased, so the maternal serum levels will be reduced [181].

Data on vedolizumab exposure during pregnancy are more limited than on IFX, and it seems that it is linked to a higher percentage of miscarriage, but because of the scarce data, a conclusion can not be made, as the patients included frequently experienced a flare of IBD during pregnancy [199,200] (Table 2).

The placental transfer of vedolizumab is lower than in anti-TNF agents, resulting in lower drug levels in the cord blood compared to maternal levels at the time of delivery [201]. It also seems that the clearance of vedolizumab is higher. Flanagan et al. described that at 15 weeks postpartum, the molecule was not detected in infant serum [181].

**Table 2:** Pregnancy outcomes in females taking vedolizumab during pregnancy.

Study	Pregnancies	Live Births	Spontaneous Abortion	Congenital Anomalies	Premature Deliveries	Low Birth Weight	Caesarian Section	Type of Congenital Anomalies
Moens et al. [199]	24	23	1	3	4	1	5	Congenital pulmonary valve stenosis Hip dysplasia Hirschprung
Bar et al. [202]	24	19	5	1	5	0	0	Hypotiroidism congenital
Mitrova et al. [201]	24	22	2	0	0	1	9	
Julsgaard et al. [203]	4	4	0	0	0	0	0	
Sheridan et al. [204]	1	0	0	0	0	0	0	
Flanagan et al. [205]	5	0	0	1	0	0	4	Hip dysplasia

#### 4.2.8. Ustekinumab 

Ustekinumab is a human monoclonal antibody that binds to the p40 subunit of IL-12 and IL-23 and inhibits their activity.

Ustekinumab has not been extensively studied in pregnant women with IBD. The literature mainly provides case reports and rare case studies. Martin et al. evaluated the effect of ustekinumab in pregnant macaques and concluded that it is safe during pregnancy, both for mother and fetuses, and the clearance in infant serum was slow; low levels were found in infant serum at 120 days post-birth [206] (Table 3).

The placental transfer of ustekinumab seems to have a similar pattern with anti-TNF drugs, with levels in the cord blood correlated and exceeding those in maternal blood but without correlation between cord drug level and the interval between the last dose and delivery [201].

Klenke et al. described a case in which the mother was taking ustekinumab during pregnancy and breastfeeding without any consequences on the mother or fetus and infant development at 1 year [207]. The abortion rate is slightly higher than the general population but without statistical significance.

#### 4.2.9. Tofacitinib

Tofacitinib is an oral Janus kinase inhibitor for the treatment of UC, rheumatoid arthritis and psoriasis. Animal studies observed an increased risk of miscarriage and teratogenic effects [48]. A human study that included 47 women with rheumatoid arthritis and psoriasis that were exposed mainly during the first trimester of pregnancy at Tofacitinib reported 25 healthy newborns, seven spontaneous abortions, eight medical terminations and just one congenital malformation–pulmonary valve stenosis, thus showing that exposure to tofacitinib during conception may not be associated with an increased risk to the fetus [213]. The perinatal maternal or paternal UC patients’ exposure to tofacitinib appears to not increase adverse pregnancy outcomes in a small study [214]. 

#### 4.2.10. Antibiotic Therapy

Metronidazole and ciprofloxacin are indicated in IBD patients to treat pouchitis, perianal and intra-abdominal abscesses and fistulae.

Animal studies showed that metronidazole during pregnancy might have a carcinogenic effect [215]. In humans, large studies and meta-analyses found that the use of metronidazole in all trimesters was not associated with adverse pregnancy outcomes or congenital malformations [216,217,218], while one-case control study detected an increased risk of cleft deformities [219], and another one suggested an increased risk of premature birth [220].

Regarding the use of ciprofloxacin during pregnancies, studies found no increased risk of major malformations and adverse pregnancy outcomes [221,222,223], but there is a theoretical risk of fetal musculoskeletal development impairment [215]. ECCO guidelines state that metronidazole and ciprofloxacin should be avoided in the first trimester [36].

Penicillins have not been shown to determine fetal malformation or adverse pregnancy outcomes and are considered the first-line antibiotic therapy in pregnancy [217].

## 5. The Impact of IBD on the Neonatal Period

### 5.1. Vaccination

Studies have demonstrated that infants born to mothers treated with anti-TNF can have measurable drug concentration for up to 12 months [182,224]. The persistence of anti-TNF at detectable concentrations leads to European and North American guidelines to recommend delaying vaccination with live vaccines, including Bacille Calmette–Guérin, rotavirus, oral polio, measles, mumps, rubella and chickenpox vaccine, until 6 months post-delivery and European Medicines Agency for at least 12 months after delivery [225]. Data on the safety of administering a live vaccine to these infants are sparse, with at least one reported fatality due to disseminated BCG infection of an infant vaccinated 3 months after being born from a mother that had taken infliximab during pregnancy [224], but recent studies showed no vaccine-related severe adverse effects after BCG vaccination in the first 6–12 months after delivery [226,227,228].

Regarding rotavirus vaccination, there is a paucity of data that suggests that it may be safe, but further studies are needed in order to make a recommendation [229]. In order to be effective, the vaccine should be administered by 15 weeks of age, but the British society of gastroenterology and AGA recommend not to be administered at all for children exposed to anti-TNF in utero [10,230].

Vaccines for chickenpox, measles, mumps and rubella are administered at the age of 1 year and can be administered even while the infant is breastfed [10].

Other types of vaccines, except live vaccines, are safe with good responses for children exposed to anti-TNF during pregnancy [231].

### 5.2. Treatment of IBD during Breastfeeding

Studies show that in the post-partum period, 44–94.6% of women initiate breastfeeding at delivery [232,233,234,235], but with the passage of time, they discontinue more often due to perceived insufficient milk production and concerns of infant medication exposure through breast milk [233]. Studies on the impact of breastfeeding on IBD activity are inconsistent, some suggesting no impact [36,236], while others describe a possible protective effect against disease flare in the first year postpartum [234,235] and also a reduced risk of early onset IBD in children [237,238].

#### 5.2.1. 5-ASA

5-ASA is poorly excreted into breastmilk, Silverman et al. calculated that a baby consuming an average quantity of breastmilk (150 mL/kg/day) will ingest 0.0006–0.006 mg/kg of 5-ASA, which is a level considered safe [239]. Sulfapyridine moiety of sulfasalazine is excreted into breastmilk [240], and sulfasalazine has been associated with bloody diarrhea, fever and vomiting in breastfed infants [241], therefore non-sulfasalazine formulations of 5-ASA are preferred in lactating women. ECCO guidelines consider breastfeeding safe while exposed to aminosalicylates [36].

#### 5.2.2. Corticosteroid

Corticosteroid excretion into breast milk is relatively low but dose-dependent [242]. Ost et al. calculated that an infant consuming 100 mL/kg/day breastmilk would ingest <0.1% of 80 mg/day dose of prednisolone [243]. Women who are administered >20 mg/day of prednisolone may be advised to delay breastfeeding for four hours after administration to reduce neonatal exposure [36,244]. Prednisolone is the preferred formula because it reaches lower breast milk levels than prednisone. Breastfed infants from mothers taking corticoids, up to 40 mg/day of prednisone, seem to have no adverse effects [245].

#### 5.2.3. Thiopurine 

Thiopurine excretion into the breast milk is low; Christensen et al. calculated that an infant consuming 150 mL/kg/day breast milk from a mother taking a therapeutic dose would ingest <1% of the adult dose mercaptopurine. The thiopurine peak level is reached within four hours of drug ingestion and declines 10% of this level two hours later [246]. Multiple studies of women with IBD that have taken thiopurines while breastfeeding found no adverse effect on infant development [247,248,249].

#### 5.2.4. Methotrexate

Methotrexate excretion into breast milk is limited, but low levels can be detected for 7 days after drug administration. Data are insufficient on infant outcomes, and it is recommended to avoid administrating methotrexate in lactating women [10,27,250].

#### 5.2.5. Cyclosporine 

May be excreted in breast milk at variable concentrations [251]. Although no adverse effects had been identified for the infant exposed to breast milk of a mother taking cyclosporine [171,252], and breastfeeding is not discouraged [10,250], there are some concerns regarding the potential carcinogenetic effect [253].

#### 5.2.6. Anti-TNF Medications 

Infliximab and adalimumab have very low levels detectable in breast milk, without adverse effects on the development and rates of infection in the infant, so numerous guidelines, including ECCO, have found it to be acceptable during breastfeeding [36,254]. For children of IBD mothers treated with anti-TNF, similar growth and psychomotor development and no difference in rates of infection and allergy were observed [255,256]. Children born to mothers treated during pregnancy with a combination of thiopurine and anti-TNF had an increased risk of serious infection during the first year of life [257].

#### 5.2.7. Ustekinumab

There are scarce data that study the ustekinumab breast milk levels in IBD patients. Studies on macaques show a very small amount of ~ 1/1000th of the serum blood concentration of ustekinumab in breast milk [206]. Matro et al. demonstrated low ustekinumab levels in breast milk in four of six women, up to a maximum of 1.57 μg/mL [258]. Based on very limited data, the ow concentration of ustekinumab in breast milk are unlikely to cause immunosuppression in infants; thus, breastfeeding is probably safe, but more studies are needed before solid recommendations can be formulated.

#### 5.2.8. Vedolizumab

In the Moens study, 12 babies out of 23 were breastfed and were vaccinated without allergic or adverse reactions to vaccination reported. No serious infections or malignancies were reported during the median follow-up time of 23 weeks [199].

#### 5.2.9. Tofacitinib

There are no human studies that report outcomes of breastfeeding with tofacitinib. Due to its small molecule, it is assumed to be excreted into breast milk, as studies have shown it is present in rat milk at twice the concentration of that in the serum [254]. The recommendation is not to breastfeed for 18 h or 36 h after immediate or delayed-release tofacitinib ingestion, respectively [48].

#### 5.2.10. Antibiotics

Metronidazole and ciprofloxacin are excreted into breast milk, and ECCO guidelines recommend avoiding them during the breastfeeding period [36]. Low levels of metronidazole and its metabolite have been detected in the serum of breastfed infants, with an estimated breastfed infant consuming <10% of the therapeutic infant dose per day, which may induce diarrhea and candida in rare cases [259]. The American Academy of Pediatrics Committee on Drugs recommends discontinuing breastfeeding for 24 h after single-dose maternal treatment [260].

Ciprofloxacin was also detected at low levels in milk [261], and withholding breastfeeding for 4 h after administration was suggested in order to decrease the exposure of the infant to medicine in breastmilk [262].

## 6. Discussion and Conclusions

The increasing incidence of young IBD patients in the last years, affecting them during their fertile period, raises important questions regarding conception, pregnancy and breastfeeding. These questions are not only raised among patients but also among general practitioners, gynecologists and gastroenterologists. Consequently, practice guidelines and IBD programs have been implemented to improve management and provide accurate information. Fertility is mostly affected in CD due to active disease flares that lead to local inflammation and scarring that may also affect the reproductive system [40,43].

VC is mostly due to the poor understanding of the possible evolution and treatment option of the disease from IBD patients. This decision may have a negative impact on the patient’s quality of life, leading to depression.

Active IBD is associated with increased rates of low-birth weight, preterm birth, spontaneous abortion, early gestational age and stillbirths [105], so controlling the disease activity both before and during pregnancy is a turning point. In some countries, the development of dedicated IBD-pregnancy clinics that can give counseling and education by trained IBD physicians and obstetricians seems to lead to better control of IBD flares during pregnancy and improve birth outcomes [28].

Treatment continuity during pregnancy is crucial to maintain disease control and avoid flares. 5-ASA, thiopurines and anti-TNF agents are considered safe during pregnancy with a low risk of adverse outcomes. Regarding corticoid treatment, most authors consider that the benefit outweighs the risks, but there are some studies that identified an increased risk of cleft lip and palate secondary to the administration of high doses. Methotrexate and tofacitinib should be avoided due to their teratogenic effects. Newer agents, such as ustekinumab and vedolizumab, seem safe to administer during pregnancy, but more studies should be conducted.

A significant number of IBD patients choose not to breastfeed, mostly due to fear of medication transfer in breast milk. Although data on safety are sparse, most IBD medication seems safe during breastfeeding. Women using corticoid therapy at doses over 20 mg should delay breastfeeding for at least 4 h, and for those taking methotrexate, cyclosporin and tofacitinib, it is better to avoid breastfeeding.

## Figures and Tables

**Table 1:** Pregnancy outcomes in females taking anti-TNF during pregnancy.

Study	Pregnancies	Live Births No (%)	Spontanous Abortion No (%)	Preterm Deliveries % (No)	Congenital Anomalies No (%)	Infections	Low Bith Weight	Cesarean Section
Katushito et al. [188]	1	1	0	0	0	-	0	0
Zelinkova et al. [189]	4	4	0	0	1 (25%)	0	0	
Mahadevan et al. [190]	10	10	-	30% (3)	0	-	10% (1)	80% (8)
Casanova et al. [166]	29		9.1%	6.1%	1.7%	3%		
Correia LM [191]	2	2	0	50%(1)	0			100% (2)
Kane et al. [192]	3	3	0	33% (1)	0	0		33% (1)
Kanis et al. [184]	131	131		6.8% (9)	2.29% (3)			43.51% (57)
Katz et al. [193]	55	58		20% (11)				
Deepak et al. [194]	783		237 (30%)	3.3% (26)	1.7% (13)		1% (8)	9.83% (77)
Kiely et al. [195]	21			2 (9.52%)	0		2 (9.52%)	57.14% (12)
Lichtenstein et al. [196]	106	81 (81.8%)	16 (16.2%)	2.83% (3)	1.2% (1)			
Schnitzler et al. [197]	35	27 (77.1%)	7 (20%)	17.14% (6)	0		14.28% (5)	
Seiafi et al. [198]	133	117 (87.96%)	16 (12%)	20% (23)	1% (1)	2% (2)	16% (19)	

**Table 3:** Pregnancy outcomes in females taking ustekinumab during pregnancy.

Study	Pregnancies	Live Births	Spontaneous Abortion	Congenital Anomalies	Premature Deliveries	Low Birth Weight	Caesarian Section	Type of Congenital Anomalies
Mitrova et al. [201]	32	27	5	3	0	1	8	Hip dysplasia hydrocoele
Klenske et al. [207]	1	0	0	0	0	0	0	
Cortes et al. [208]	1	0	0	0	0	0	0	
Venturin et al. [209]	1	1	0	0	0	0	0	
Galli-Novak [210]	1	0	0	0	0	0		
Scherl et al. [211]	24	15	4	0	0	0	-	
Rowan et al. [212]	1	0	0	0	0	0	1	
Lukesova et al. [1]	1	0	0	0	0	0	1	

## Data Availability

No new data were created or analyzed in this study. Data sharing is not applicable to this article.
